# Structural and Biological Basis of Alphacoronavirus nsp1 Associated with Host Proliferation and Immune Evasion

**DOI:** 10.3390/v12080812

**Published:** 2020-07-28

**Authors:** Zhou Shen, Yiling Yang, Siqi Yang, Guangxu Zhang, Shaobo Xiao, Zhen F. Fu, Guiqing Peng

**Affiliations:** 1 State Key Laboratory of Agricultural Microbiology, College of Veterinary Medicine, Huazhong Agricultural University, Wuhan 430070, China; szhou1314@webmail.hzau.edu.cn (Z.S.); yangyilin@webmail.hzau.edu.cn (Y.Y.); siqiyang@webmail.hzau.edu.cn (S.Y.); zgx980918@webmail.hzau.edu.cn (G.Z.); vet@mail.hzau.edu.cn (S.X.); zhenfu@uga.edu (Z.F.F.); 2 Key Laboratory of Preventive Veterinary Medicine in Hubei Province, The Cooperative Innovation Center for Sustainable Pig Production, Wuhan 430070, China; 3 Department of Pathology, College of Veterinary Medicine, University of Georgia, Athens, GA 30602, USA

**Keywords:** alphacoronavirus nsp1, crystal structure, structure-function, infectious disease, cellular immune response, RNA sequence, interferon, cell cycle

## Abstract

Non-structural protein 1 (nsp1) is only characterized in alphacoronaviruses (α-CoVs) and betacoronaviruses (β-CoVs). There have been extensive researches on how the β-CoVs nsp1 regulates viral virulence by inhibiting host protein synthesis, but the regulatory mechanism of the α-CoVs nsp1 is still unclear. Here, we report the 2.1-Å full-length crystal structure of nsp1 in emerging porcine SADS-CoV and the 1.8-Å full-length crystal structure of nsp1 in the highly lethal cat FIPV. Although they belong to different subtypes of α-CoVs, these viruses all have a bucket-shaped fold composed of six β-sheets, similar to the crystal structure of PEDV and TGEV nsp1. Comparing the above four structures, we found that the structure of α-CoVs nsp1 in the same subtype was more conserved. We then selected mammalian cells that were treated with SADS-CoV and FIPV nsp1 for RNA sequencing analysis and found that nsp1 had a specific inhibitory effect on interferon (IFN) and cell cycle genes. Using the Renilla luciferase (Rluc) assay and Western blotting, we confirmed that seven representative α-CoVs nsp1s could significantly inhibit the phosphorylation of STAT1-S727 and interfere with the effect of IFN-I. Moreover, the cell cycle experiment confirmed that α-CoVs nsp1 could encourage host cells to stay in the G0/G1 phase. Based on these findings, we not only greatly improved the crystal structure data on α-CoVs nsp1, but we also speculated that α-CoVs nsp1 regulated host proliferation and immune evasion-related biological functions by inhibiting the synthesis of host proteins, thus creating an environment conducive to the virus.

## 1. Introduction

With the outbreak of severe acute respiratory syndrome coronavirus 2 (SARS-CoV-2) at the end of 2019 in Wuhan [1], the coronavirus (CoV) has received unprecedented attention. CoVs are the most complex positive-sense single-stranded RNA (ssRNA+) viruses, with a 5′-cap structure and a 3′-poly-A tail [2,3]. Genotypically and serologically, CoVs can be divided into four subfamilies: Alphacoronavirus (α-CoV), betacoronavirus (β-CoV), gammacoronavirus (γ-CoV), and deltacoronavirus (δ-CoV) [4]. α-CoV and β-CoV primarily infect the respiratory and gastrointestinal systems of mammals, while γ-CoV and δ-CoV primarily infect birds [5,6]. Within the genus α-CoV, feline infectious peritonitis virus (FIPV) is in the α-CoV 1A species, which also contains a few other closely related viruses, such as transmissible gastroenteritis virus (TGEV), porcine respiratory coronavirus (PRCV), and canine coronavirus (CCV). Other more distantly related species in the α-CoV 1B genus include swine acute diarrhea syndrome coronavirus (SADS-CoV), porcine epidemic diarrhea virus (PEDV), human coronavirus 229E (HCoV-229E), human coronavirus NL63 (HCoV-NL63), Scotophilus bat coronavirus 512, Miniopterus bat coronavirus 1, Bat coronavirus HKU8, and Bat coronavirus HKU2 [7]. As a newly discovered CoV, SADS-CoV broke out in 2017 in Guangdong Province, causing the death of approximately 25,000 piglets within a few months [8]. Furthermore, SADS-CoV re-emerged and led to the death of approximately 2000 piglets in February 2019 [9,10]. The symptoms of SADS-CoV are similar to those of other swine enteric coronaviruses, such as porcine deltacoronavirus (PDCoV) and PEDV, which poses challenges for the detection, prevention, and control of swine diarrhea pathogens [11]. FIPV is responsible for the deadly systemic immune-mediated granulomatous disease, which has a mortality rate of nearly 100% in cats [12]. Unfortunately, there are no effective measures for the prevention and control of SADS-CoV and FIPV, which have caused serious harm to the safety of human public property [13,14].

The nonstructural protein 1 (nsp1) gene was not present in all types of CoV, but only in α-CoV and β-CoV [15]. Based on a difference in the sequence analysis between α-CoV and β-CoV, nsp1 can be considered as a genus-specific marker [16]. There have been many studies on the inhibition of host protein synthesis by β-CoV nsp1, which reveals the diversity of its mechanism. Severe acute respiratory syndrome coronavirus (SARS-CoV) nsp1 inhibited host translation by binding to the 40S ribosomal subunit, and the SARS-CoV nsp1-40S ribosome complex also induced the endonucleolytic cleavage of the host mRNAs [17,18]. Unlike SARS-CoV nsp1, Middle East respiratory syndrome coronavirus (MERS-CoV) nsp1 could not stably bind to the 40S ribosomal subunit to inhibit host cell translation. Furthermore, it selectively degraded the nuclear mRNA in host cells [19,20]. Although the important functional region of α-CoV nsp1 that inhibited host protein synthesis was found [21], the specific mechanism through which α-CoV nsp1 regulated translation was not yet clear. In our previous research [22], we indicated that PEDV nsp1 could not regulate host protein synthesis through an interaction with the 40S ribosomal subunit. From a structural perspective, the critical regions of PEDV nsp1 that inhibited the host protein synthesis were different from those of SARS-CoV nsp1 [22,23], which further explained the mechanism by which α-CoV nsp1 regulated host gene expression and its specificity. A detailed host factor for regulating the translation of host proteins was not found in TGEV nsp1 [24]. Similar to β-CoV nsp1 [25,26], α-CoV nsp1 has been identified as an important virulence factor that acts by inhibiting the host protein synthesis function [21]. Research on the regulation mechanism of the host gene by α-CoV nsp1 is becoming increasingly more fascinating.

Here, we are the first to report a 1.8-Å full-length crystal structure for FIPV nsp1 and a 2.1-Å full-length crystal structure of SADS-CoV nsp1. Although SADS-CoV and FIPV belong to different α-CoV subtypes, the crystal structures of α-CoV nsp1 have a conserved β-barrel that is composed of six β-sheets, which is consistent with our previous analysis of the crystal structures of PEDV and TGEV nsp1 [21,22]. This investigation also showed that the β-barrel structure of α-CoV nsp1 was the common fold for controlling biological functions. Although β-CoV nsp1 had this fold, it only supported the function but was not contained in the critical functional region [27]. The differences in the structural roles of α-CoV and β-CoV also indicated that the mechanism was diverse in terms of the CoV nsp1 inhibition of host gene expression. However, we determined that the structure and amino acid sequence of the same subtype in α-CoV nsp1 were conserved, which was helpful for exploring the broad-spectrum biological function mechanism of α-CoV nsp1. An RNA sequencing analysis of SADS-CoV and FIPV nsp1 acting in mammalian cells showed that nsp1 not only downregulated but also upregulated the host genes. Specifically, the host gene for the compensatory related gene was upregulated and the interferon (IFN) or cell cycle genes were downregulated under the nsp1 gene that was present. Through a biochemical analysis of seven representative α-CoV nsp1s, we found that they could regulate the host IFN-I by inhibiting the phosphorylation of STAT1-S727. Furthermore, α-CoV nsp1 could regulate the host cells, causing them to stay in the G0/G1 phase. Taken together, these findings indicated that the common fold of α-CoV nsp1 was critical for inhibiting the host protein synthesis. Moreover, we explained that α-CoV nsp1 may enhance the viral virulence through the regulation of host proliferation and immune evasion.

## 2. Materials and Methods

### 2.1. Cell Lines and Viruses

Human embryonic kidney (HEK-293T), Crandell Reese Feline Kidney (CRFK), and Porcine ileum epithelial (IPI-2I) cells were obtained from the China Center for Type Culture Collection (Wuhan, China) and cultured in Dulbecco’s modified Eagle’s medium (Gibco, Waltham, MA, USA) supplemented with a 10% heat-inactivated fetal bovine serum (Invitrogen, CA, USA), 100  U/mL penicillin, and 10 μg/mL streptomycin sulfates (Beyotime, Wuhan, China) at 37 °C and 5% CO_2_ in a humidified incubator (Thermo Fisher Scientific, Runcorn, Cheshire, UK). FIPV and FIPV(91–95 sg) were rescued by our laboratory using the method described in previous research [21,28]. SADS-CoV strain CHN-GD-2017 (GenBank accession no. MH539766) was isolated from piglets with severe diarrhea in China in 2017 [29].

### 2.2. Plasmid Construction

The full-length nsp1 genes of SADS-CoV and FIPV (GenBank TM accession numbers: MH539766 and DQ010921, respectively) were amplified by the polymerase chain reaction (PCR) from virus-derived cDNA fragments and cloned separately into pET42b using homologous recombination. The forward and reverse primers contained NdeI and XhoI restriction sites, respectively. Both nsp1 proteins were expressed with an His6 tag in E. coli BL21 (DE3) cells. To achieve high expression in the HEK-293T cells, wild-type α-CoVs (SADS-CoV, MH539766; FIPV, DQ010921; TGEV, HQ462571; PRCV, KR270796; PEDV, AJP67455; HCoV-NL63, AFV53147; and HCoV-229E, CAA49377) nsp1 flanked with an N-terminal hemagglutinin (HA) tag was cloned into the pCAGGS vector using EcoRI and XhoI restriction sites. All of the recombinant expression plasmids were sequenced, and no unexpected mutations occurred.

### 2.3. Protein Purification and Crystallization

For high-quality soluble protein purification, SADS-CoV and FIPV nsp1 proteins were induced with 1 mM isopropyl-β-D-thiogalactopyranoside (IPTG) (Sigma-Aldrich Corp., St. Louis, MO, USA), and cell growth continued for an additional 18 h at 18 °C as previously described [30]. Specifically, E. coli Trans BL21(DE3) cells were lysed by passage through an AH-1500 homogenizer (ATS Engineering Inc., Shanghai, China) to obtain their periplasmic proteins. The recombinant protein was loaded onto a His TrapTM HP column (GE Healthcare, Pittsburgh, USA) that was pre-equilibrated with buffer A (pH 7.4, 20 mM Tris-HCl (Biosharp, Anhui, China), 500 mM NaCl (Biosharp)). Then, the target protein was eluted using a linear gradient between buffer A and buffer B (pH 7.4, 20 mM Tris-HCl, 500 mM NaCl, and 500 mM imidazole). Fractions that contained a protein band isolated with an Amicon Ultra (10,000 MWCO) centrifugal filter device (Millipore, Bedford, MA, USA) were pooled and loaded onto a 120-mL Superdex 200 (GE Healthcare, Pittsburgh, USA) column with buffer C (pH 7.4, 20 mM Tris-HCl, 200 mM NaCl). Finally, pET42b-FIPV nsp1 and pET42b-SADS-CoV nsp1 were concentrated to 10.23 and 9.85 mg/mL, respectively. All the purification procedures were performed at 4 °C to prevent degradation.

A crystallization screening was performed by the sitting drop vapor diffusion method at 20 °C. According to the initial conditions, which we optimized for crystallization, the best SADS-CoV nsp1 crystal was obtained by vapor diffusion in sitting drops consisting of 1 μL of a reservoir solution (0.2 M ammonium citrate dibasic (Biosharp, Anhui, China), 23% *w*/*v* polyethylene glycol 3350 (Hampton Research, California, USA)), and 1 μL of protein solution (8.5 mg/mL in 20 mM Tris-HCl and 200 mM NaCl, pH 7.4). The FIPV nsp1 protein crystals appeared within 1 day at 20 °C in 0.2 M ammonium citrate dibasic and 20% *w*/*v* polyethylene glycol 3350.

### 2.4. Data Collection and Structure Determination

X-ray diffraction data were collected at beamline BL17U of the Shanghai Synchrotron Radiation Facility. The SADS-CoV and FIPV nsp1 structures were solved using the molecular replacement method, and the initial phases were calculated using the molecular replacement program from the PHENIX software suite. Manual model rebuilding was performed using COOT [31], and the model was then refined in the PHENIX software suite [32]. Structural figures were drawn using PyMOL [33]. The RMSD values were analyzed using PDBeFold (http://pdbe.org/fold/). The amino acid (aa) sequences of the α-CoV nsp1 proteins were aligned using the software program ClustalW2 and visualized with the ESPript 3 server (http://espript.ibcp.fr). Evolutionary relationships were evaluated using the MEGA software [34]. Phasing and refinement details are presented in Table 1.

### 2.5. Reporter Assay and Western Blot Analysis

HEK-293T cells at ~80% confluence were co-transfected with a pRL-IFN-β or pRL-ISRE plasmid encoding the Rluc reporter gene downstream of the IFN-β or ISRE promoter together with the corresponding protein plasmids. At 8 h post-transfection, the cells were induced with either 2.5 μg of poly (I:C) (Sigma) or 500 U/mL IFN-α (Sigma). After an incubation period of 16 h, the cells were harvested and lysed in 120 μL of a Reporter Lysis buffer (Promega, Wisconsin, USA). A 10-μL aliquot of lysate was used to measure the luciferase activity as described by the manufacturer (Promega, WI, USA).

The protein expression was analyzed via Western blotting, and the proteins were visualized using an anti-HA antibody (Ab; ProteinTech, Wuhan, China), STAT1 antibody (Ab; Abclonal, Wuhan, China), STAT1-Y701 antibody (Ab; Abclonal, Wuhan, China) or STAT1-S727 antibody (Ab; Abclonal, Wuhan, China). The expression of glyceraldehyde-3-phosphate dehydrogenase (GAPDH) was assessed with an anti-GAPDH monoclonal Ab (mAb; ProteinTech, Wuhan, China) to confirm equal protein loading. The gray scale values of the protein bands were analyzed with ImageJ (http://rsbweb.nih.gov/ij/). Reporter assay and Western blot assay datasets are presented as the means ± standard deviations (SDs) of at least three independent experiments.

### 2.6. Indirect Immunofluorescence Assay (IFA)

To examine the expression of α-CoVs nsp1 in HEK-293T, the cells (2 × 10^5^/well) seeded in 24-well plates (Nest, Jiangsu, China) were transfected with the corresponding plasmids for 24 h. Subsequently, the cells were washed three times with 500 mL of a phosphate-buffered saline (PBS) and fixed with 4% paraformaldehyde (BOSTER, California, USA) for 15 min and 0.2% Triton X-100 (Biosharp) for 10 min. Afterward, the cells were blocked with bovine serum albumin (5%) for 1 h and then incubated with mAbs against the HA protein for 1 h. Following three washes with 500 mL of PBS, a fluorescence isothiocyanate (FITC)-conjugated goat anti-mouse IgG (Santa Cruz Biotechnology, USA) was added and incubated for 1 h. The cell nuclei were stained with 0.01% 4′,6-diamidino-2-phenylindole (DAPI) (Sigma-Aldrich Corp., St. Louis, MO, USA) for 15 min at room temperature and visualized using an inverted fluorescence microscope (Olympus IX73).

### 2.7. Quantitative Analysis of Rluc mRNA

To determine the effect of SADS-CoV and FIPV nsp1 on the expression of the host genes, including IRF9, CDKN1A, ISG15, E2F2, and STAT1, the HEK-293T cells (1 × 10^6^/well) in 6-well plates (Nest, Jiangsu, China) were transfected with 2.5 μg of empty vector or nsp1 expression plasmid. After 24 h, the total cellular RNA was extracted from the transfected cells with a TRIzol reagent (Invitrogen, Carlsbad, CA, USA). To further examine the selected genes in susceptible cells, FIPV nsp1 and SADS-CoV nsp1 were transfected into CRFK and IPI-2I cells, respectively. The RNA was then reverse-transcribed into cDNA with avian myeloblastosis virus reverse transcriptase (TaKaRa, Japan). Then, the cDNA was used as the template in a SYBR green quantitative real-time PCR (RT-qPCR) assay (Applied Biosystems, Foster City, CA, USA). Moreover, RT-qPCR experiments were performed in triplicate. The mRNA expression levels were normalized to the GAPDH level. The specific primer sequences used in this study are listed in Table 2. RT-qPCR datasets are presented as the means ± SD of at least three independent experiments.

### 2.8. RNA Sequence Analysis

The differential expression of mRNAs in the sample of HEK-293T cells treated with FIPV nsp1, SADS-CoV nsp1 or pCAGGS was determined using DESeq, with *p* < 0.05 and fold change (FC) ≥ 2. The IFN-related genes (ISG15, SOCS1, IFI6, OAS1, GBP1, EIF2AK2, IFIT2, STAT1, IRF8, CIITA, CXCL10, and IRF9) and cell cycle-related genes (CDKN1A, E2F2, ORC1, SFN, MDM2, and TGFB1) were significantly downregulated in the FIPV nsp1 and SADS-CoV nsp1 groups. Additionally, five of the mRNAs (STAT1, IRF9, ISG15, CDKN1A, and E2F2) were selected for validation by RT-qPCR.

### 2.9. DNA Content Analysis

The HEK-293T cells (1 × 10^6^/well) were seeded into 6-well plates and incubated overnight at 37 °C in a 5% CO_2_ incubator. The cells were then transfected with 2.5 μg of empty vector or nsp1 expression plasmid for 24 h. To verify the effect of the virus on the susceptible cell cycle, we infected CRFK and IPI-2I with 0.1 Multiplicity of infection (MOI) FIPV and SADS-CoV for 12 and 24 h, respectively. The cells were collected and fixed in 75% ethanol overnight at 4 °C. In accordance with the manufacturer’s instructions (Cat no. FXP0211, 4A Biotech Co., Ltd, Shanghai, China), the fixed cells were incubated with a 50 ng/mL PI staining solution and 0.1 mg/mL RNase A for 30 min in the dark at 37 °C, and fluorescence intensity was measured with a BD FACSCalibur (BD Biosciences, San Jose, CA, USA) [35]. Finally, the results were analyzed using the ModFit software (BD Biosciences, San Jose, CA, USA). Cell cycle datasets are presented as the means ± SD of at least three independent experiments.

### 2.10. Growth Curves of Viruses

CRFK cells were infected with FIPV or FIPV (91–95 sg) at a multiplicity of infection (MOI) of 0.1 in 6-well plates. Subsequently, the supernatants of the infected cells at 6, 12, 18, 24, 30, and 36 h post-infection were collected and stored at −80 °C [24]. The viral titers at each time point were determined by TCID_50_.

### 2.11. Protein Structure Accession Numbers and Statistical Analysis

The coordinates and structural characteristics of SADS-CoV and FIPV nsp1 have been deposited in the Research Collaboratory for Structural Bioinformatics (RCSB) Protein Data Bank (PDB) under accession codes 6LPA and 6LP9, respectively. The statistical calculations were performed with the GraphPad Prism software (GraphPad Software Inc., La Jolla, USA) and SPSS 21.0 (IBM-SPSS Inc., Chicago, IL, USA). Student’s t-test or the Kruskal-Wallis test was performed for normally distributed data to determine statistical significance. Statistical details of experiments are described in the figure legends. A p-value less than 0.05 is considered statistically significant.

## 3. Results

### 3.1. Overall Structures of SADS-CoV and FIPV nsp1

**Figure 1:**
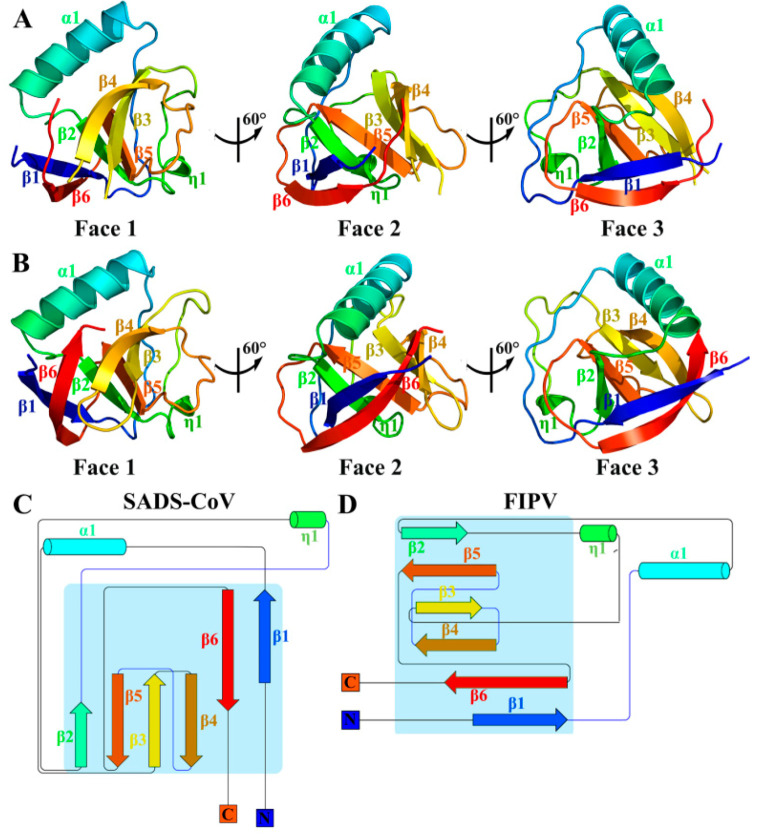
Overall architecture of swine acute diarrhea syndrome coronavirus (SADS-CoV) and feline infectious peritonitis virus (FIPV) non-structural protein 1 (nsp1). (**A**,**B**) Faces 1 to 3 of the SADS-CoV and FIPV nsp1 structures are illustrated in rainbow colors from the N terminus (blue) to the C-terminus (red). (**C**,**D**) Topology diagram of SADS-CoV and FIPV nsp1, with secondary structural elements sequentially numbered and colored from N to C as described for panels A and B. α-helices are indicated by cylinders, and β-strands are indicated by arrows. Panels A and B were produced using PyMOL (Delano Scientific), and the topology diagram was produced using Pro-origami.

The crystals of the full-length SADS-CoV nsp1 and FIPV nsp1 (including the N-terminal His6 tag) were obtained, and data of sufficient quality were collected. The structures of SADS-CoV nsp1 and FIPV nsp1 were determined by molecular replacement using the structures of PEDV nsp1 (Protein Data Bank [PDB] code 6XBC) and TGEV nsp1 (PDB code 6IVC) as the search template, respectively [21,22]. The SADS-CoV nsp1 and FIPV nsp1 structures were refined to 2.1- and 1.8-Å resolutions, respectively. Their space groups are P2_1_2_1_2_1_ and C121, respectively. With the exception of the regions encompassing amino acids 105 to 110 of SADS-CoV nsp1 and 106 to 110 of FIPV nsp1, all the residues of both nsp1 proteins can be built using the final models (Figure 1A,B). Each asymmetric unit of SADS-CoV nsp1 and the FIPV nap1 structures contained two and four subunits, respectively.

The crystal structures of SADS-CoV nsp1 and FIPV nsp1 reveal that their monomers contain six antiparallel β-strands and two α-helices (Figure 1C,D). Combined with the crystal structures of PEDV nsp1 and TGEV nsp1 (PDBs code 6XBC and 6IVC, respectively), the six β-strands forming a β-barrel fold are considered as a common fold in α-CoV nsp1.

### 3.2. Sequence Analysis of Alphacoronaviruses nsp1

**Figure 2:**
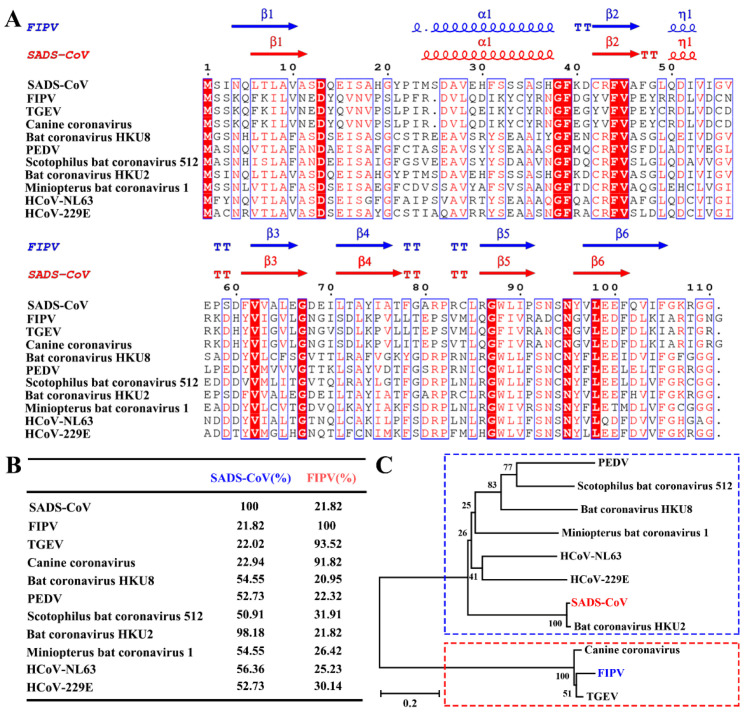
Amino acid sequence alignments and phylogenetic relationships of nsp1 proteins from seven betacoronaviruses (α-CoVs). (**A**,**B**) Sequence alignment of α-CoV nsp1 homologs to SADS-CoV nsp1 and FIPV nsp1. The following sequences from GenBank were used to create the sequence alignment (marked as labelled, GenBank TM accession number): SADS-CoV, MH539766; FIPV, DQ010921; transmissible gastroenteritis virus (TGEV), HQ462571; Canine coronavirus, KP981644; Bat coronavirus HKU8, NC_010438; porcine epidemic diarrhea virus (PEDV), AJP67455; Scotophilus bat coronavirus 512, NC_009657; Bat coronavirus HKU2, NC_009988; Miniopterus bat coronavirus 1, EU420138.1; HCoV-NL63, AFV53147; and HCoV-229E, CAA49377. The secondary structural elements of SADS-CoV nsp1 (red) and FIPV nsp1 (blue) are marked at the top of the alignment (helices with squiggles, β-strands with arrows, and turns with TT letters). Residues boxed in red are completely conserved. The sequences were aligned with ClustalW2, and the figure was prepared with ESPript3.0. (**C**) Nsp1 protein sequences from α-CoVs were analyzed by the neighbor-joining method using the maximum likelihood algorithm in the MEGA package. Each bootstrap value was determined by 1000 replicates. The scale bar represents the relationship between the line lengths and sequence dissimilarities.

In our previous study [22], we showed that the core structure of α-CoVs and β-CoVs nsp1 is similar, but the amino acid sequence homology is low. Although α-CoV nsp1 has a common fold, the homology of its amino acid sequence is not clear. To determine the relationship among α-CoV nsp1 sequences, we selected and aligned representative sequences using the ClustalW2 software (Figure 2A), which were HCoV-229E, HCoV-NL63, PEDV, TGEV, SADS-CoV, CCV, Scotophilus bat coronavirus 512, Miniopterus bat coronavirus 1, Bat coronavirus HKU8, Bat coronavirus HKU2, and FIPV nsp1.

Compared with the amino acid sequence homology of β-CoV nsp1, the amino acid sequence homology of α-CoV nsp1 is higher. The α-CoV nsp1 sequence alignment showed some highly conserved residues, including Asp13, Gly38, Phe39, Phe44, Val45, Val62, Gly68, Gly87, Asn95, and Leu98, some of which were mentioned in our previous report [22]. Moreover, some areas were shown to be relatively conserved, including amino acid motifs 8–10, 16–18, 26–28, 37–39, 61–63, 87–90, and 97–103. Although similarities were observed among the different α-CoVs nsp1 proteins, some differences were obvious (Figure 2B). Specifically, despite the homology among SADS-CoV nsp1 with PEDV nsp1, HCoV-229E nsp1, Scotophilus bat coronavirus 512 nsp1, Miniopterus bat coronavirus 1 nsp1, Bat coronavirus HKU8 nsp1, Bat coronavirus HKU2 nsp1, and HCoV-NL63 nsp1 being very high, ranging from 50% to 100%, the homology among FIPV nsp1, CCV nsp1, and TGEV nsp1 was very low, at only approximately 22%. Furthermore, the amino acid sequence of FIPV nsp1 has a low shared sequence identity with PEDV nsp1, SADS-CoV nsp1, HCoV-229E nsp1, Scotophilus bat coronavirus 512 nsp1, Miniopterus bat coronavirus 1 nsp1, Bat coronavirus HKU8 nsp1, Bat coronavirus HKU2 nsp1, and HCoV-NL63 nsp1, ranging from 20% to 35%. However, the amino acid sequence of FIPV nsp1 is extremely conserved compared with those of CCV nsp1 and TGEV nsp1. Based on their distances on the evolutionary tree (Figure 2C), α-CoVs can be classified into two categories, with FIPV, TGEV, and CCV in one category and SADS-CoV, PEDV, HCoV-229E, Scotophilus bat coronavirus 512, Miniopterus bat coronavirus 1, Bat coronavirus HKU8, Bat coronavirus HKU2, and HCoV-NL63 in the other. Taken together, these results demonstrate that α-CoVs nsp1 have two distinct clades (A and B), namely, α-CoV 1A and α-CoV 1B, which is consistent with previous helicase analysis results [36].

### 3.3. Structural Comparisons of Alphacoronavirus 1A and 1B nsp1 Proteins

**Figure 3:**
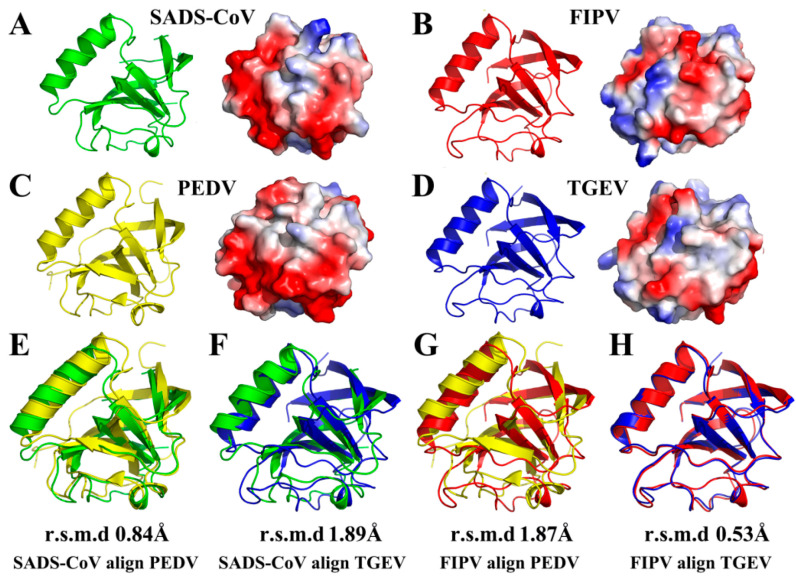
Structural comparisons of SADS-CoV nsp1, FIPV nsp1, PEDV nsp1 (PDB code 5XBC), and TGEV nsp1 (PDB code 6IVC). (**A**) Conformational states of the SADS-CoV nsp1 monomer. (**B**) Conformational states of the FIPV nsp1 monomer. (**C**) Conformational states of the PEDV nsp1 monomer. (**D**) Conformational states of the TGEV nsp1 monomer. SADS-CoV nsp1 is shown in green, FIPV nsp1 is shown in red, PEDV nsp1 is shown in yellow, and TGEV nsp1 is shown in blue. (**E**) Structural alignment of nsp1 proteins from SADS-CoV (green) and PEDV (PDB code 5XBC, yellow). (**F**) Structural alignment of nsp1 proteins from SADS-CoV (green) and TGEV (PDB code 6IVC, blue). (**G**) Structural alignment of nsp1 proteins from FIPV (red) and PEDV (PDB code 5XBC, yellow). (**H**) Structural alignment of nsp1 proteins from FIPV (red) and TGEV (PDB code 6IVC, blue). The RMSDs following alignment by Cα atoms are listed.

In our previous studies [21,22], we resolved two full-length α-CoV nsp1 structures, TGEV nsp1 (PDB ID 6IVC) and PEDV nsp1 (PDB ID 5XBC). Here, we analyzed a 1.8-Å full-length crystal structure of FIPV nsp1 and a 2.1-Å full-length crystal structure of SADS-CoV nsp1. Interestingly, FIPV and TGEV belong to α-CoV 1A, while SADS-CoV and PEDV belong to α-CoV 1B. Therefore, we could judge the characteristics of the same subtype and different subtypes of α-CoV by structural comparison. This comparison showed that the overall structure of FIPV nsp1 is conserved relative to that of TGEV nsp1 (with an RMSD of 0.53 Å) (Figure 3H), but it has several differences compared with PEDV nsp1 (with an RMSD of 1.87 Å) (Figure 3G). Similarly, structure comparison showed that the overall structure of SADS-CoV nsp1 is conserved relative to that of PEDV nsp1 (with an RMSD of 0.84 Å) (Figure 3E), but it has several differences relative to TGEV nsp1 (with an RMSD of 1.89 Å) (Figure 3F). From the surface electrostatics, the structure of FIPV nsp1 was more similar to that of TGEV nsp1 and the structure of SADS-CoV nsp1 was more similar to that of PEDV nsp1 (Figure 3A–D). Specifically, SADS-CoV nsp1 and PEDV nsp1 have a wider distribution of negative charges on the surface than FIPV nsp1 and TGEV nsp1. Considering these data together, the structures of the same sub-type α-CoV nsp1s show greater similarity than the different sub-type α-CoV nsp1s.

The overall structure of α-CoVs nsp1 shared the same fold, with a characteristic six-stranded β-barrel formed by β1 relative to β6, β2 relative to β5, and β3 relative to β4. However, the three-dimensional alignments also revealed several differences in the structures, including in the lengths and rotations of the loops (Figure 3A–D). Specifically, the most obvious difference between the structures of PEDV nsp1 and the other α-CoV nsp1s is the electron density at the C-terminus. Compared with the unclear electron density at the C-terminal end of the other α-CoV nsp1s, the C-terminus of PEDV nsp1 is stabilized by β4, and the electron density can be observed clearly. The structures of the same sub-type α-CoVs nsp1 also exhibit several differences. For SADS-CoV nsp1 and PEDV nsp1, the length of the β6 is significantly shorter in SADS-CoV nsp1. Additionally, there are also substantial differences in the loops, especially those located at residues 11 to 22 in SADS-CoV nsp1. Furthermore, the structures of α-CoV 1A nsp1s and α-CoV 1B nsp1s also exhibit obvious differences in some loops, especially those located at residues 52 to 59 in SADS-CoV nsp1. The overall structure of FIPV nsp1 and TGEV nsp1 is highly conserved, which shows that the differential amino acids (Asn2, Cys35, Asp71, Ser91, and Phe102) in TGEV nsp1 and FIPV nsp1 will not affect the stability of their overall structure (Figure 2A). Taken together, these data indicate that although the structures of α-CoV nsp1s are similar overall, there are also differences.

### 3.4. RNA Sequences in HEK-293T Cells Transfected with SADS-CoV or FIPV nsp1

**Figure 4:**
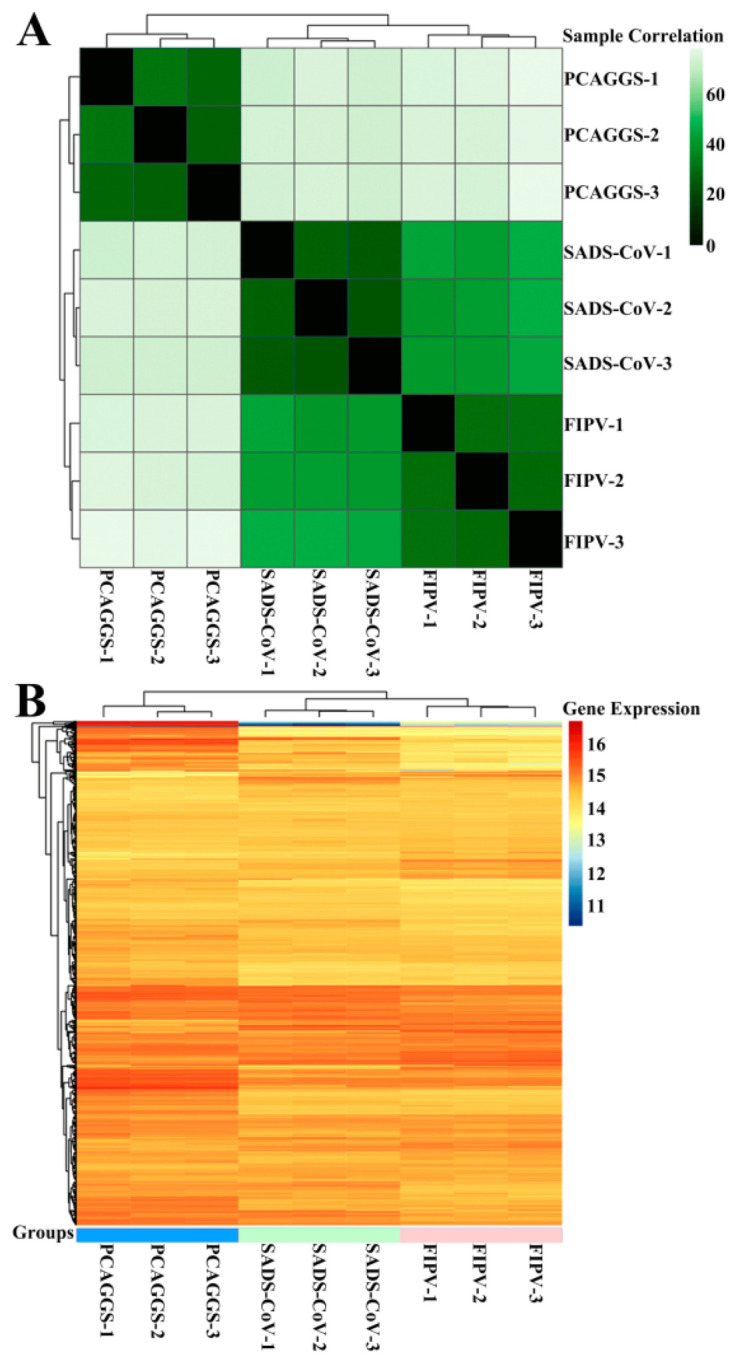
RNA sequences in HEK-293T cells transfected with SADS-CoV nsp1 or FIPV nsp1. (**A**) The correlations between samples are shown. Horizontal and vertical axes represent each sample, and different colors represent different correlation coefficients. (**B**) The abscissa represents the sample name and the clustering results for the sample, and the ordinate represents the differentially expressed genes and the clustering results for the genes. Different columns represent different samples, and different rows represent different genes. Colors represent the log2 (normalized counts) values of the expression levels for the genes in the samples.

**Figure 5:**
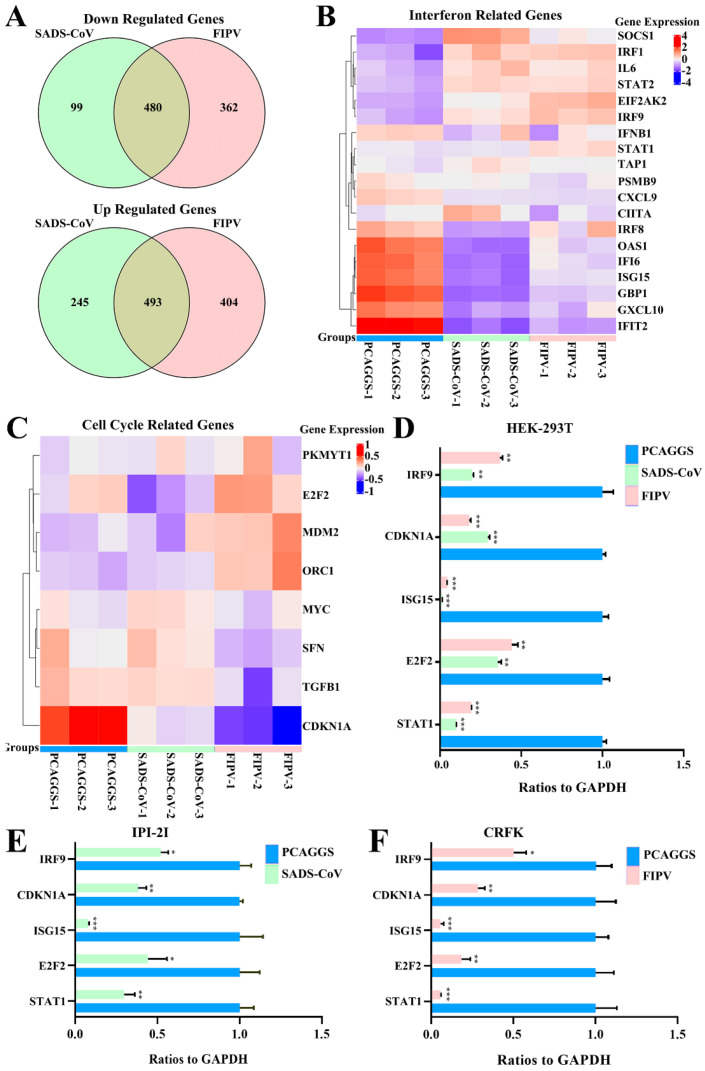
RNA sequence analysis showed that SADS-CoV nsp1 and FIPV nsp1 downregulated IFN genes and cell cycle genes. (**A**) A Venn analysis was used to screen differentially expressed mRNAs in SADS-CoV and FIPV nsp1 samples compared with their corresponding controls. Among the downregulated genes, 480 common mRNAs and 99 specific mRNAs in the group of SADS-CoV nsp1 and 362 specific mRNAs in the group of FIPV were identified (up). Among upregulated genes, 493 common mRNAs and 245 specific mRNAs in the group of SADS-CoV nsp1 and 404 specific mRNAs in the group of FIPV were identified (down). (**B**) Differentially expressed IFN-related genes, consisting of 19 downregulated genes, are displayed in the heat map. (**C**) Differentially expressed cell cycle-related genes, consisting of eight downregulated genes, are displayed in the heat map. (**D**) Quantitative real-time PCR analysis of selected mRNAs (STAT1, IRF9, ISG15, CDKN1A, and E2F2) in HEK-293T cells. (**E**) Quantitative real-time PCR analysis of selected mRNAs (STAT1, IRF9, ISG15, CDKN1A, and E2F2) for SADS-CoV nsp1 treated in IPI-2I cells. (**F**) Quantitative real-time PCR analysis of selected mRNAs (STAT1, IRF9, ISG15, CDKN1A, and E2F2) for FIPV nsp1 treated in CRFK cells. The data are presented as the means ± standard deviation (SD) (n = 3). Asterisks indicate statistical significance as determined by Student’s t-test. *, *p* < 0.05; **, *p* < 0.01; and ***, *p* < 0.001.

**Figure 6:**
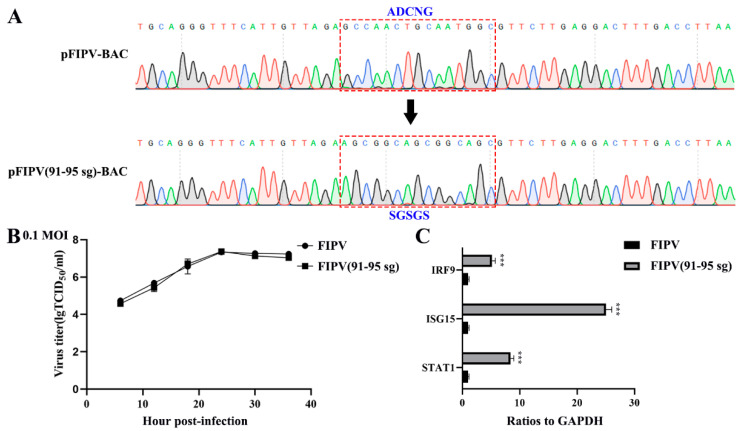
Quantitative real-time PCR analysis of FIPV or FIPV (91–95 sg) infectious clone in CRFK cells. (**A**) Sequence analysis of the targeted mutation area between pFIPV-BAC (pBeloBAC11) and pFIPV (91–95 sg)-BAC by RT-PCR sequencing. (B) Growth curves with the wild-type viruses FIPV and FIPV (91–95 sg) with an original multiplicity of infection (MOI) of 0.1. (**C**) Quantitative real-time PCR analysis of selected mRNAs (STAT1, IRF9, and ISG15) for FIPV or FIPV (91–95 sg) treated in CRFK cells. The data are presented as the means ± standard deviation (SD) (*n* = 3). Asterisks indicate statistical significance as determined by Student’s t-test. ***, *p* < 0.001.

Although the overall structures of α-CoVs 1A and 1B display several differences according to previous reports [21,25], they both significantly inhibit the synthesis of host genes in a broad-spectrum manner. However, α-CoVs nsp1 inhibiting host gene expression is a non-specific inhibition [21,24], the mechanism by which α-CoV nsp1 exerts this regulatory effect remains unknown. To determine whether α-CoV nsp1 proteins share some common mechanisms, we selected one α-CoV 1A nsp1 (FIPV nsp1) and one α-CoV 1B nsp1 (SADS-CoV nsp1) as representatives for RNA sequencing in HEK-293T cells. To evaluate the correlation among repetitive samples, we calculated the reliability of the experiments to determine whether the selected samples were reasonable. The close correlation coefficients showed that the quality of the samples was very good (Figure 4A). Depending on the relative expression levels detected in the samples, the gene expression was determined to be either upregulated or downregulated (Figure 4B). The sample analysis demonstrated numerous upregulated and downregulated genes; 1003 upregulated genes and 612 downregulated genes were found in the SADS-CoV nsp1 samples, and 1181 upregulated genes and 896 downregulated genes were found in the FIPV nsp1 samples. In an effort to elucidate a shared mechanism between α-CoVs nsp1 proteins, we performed an intersection analysis of the up- and downregulated genes in the SADS-CoV nsp1 and FIPV nsp1 samples, and the Venn diagram revealed 480 identical downregulated genes and 493 identical upregulated genes (Figure 5A). Moreover, many of the upregulated genes are related to metabolism, such as the compensatory genes, indicating a compensatory effect of the host under the action of the α-CoV nsp1 proteins. However, we paid more attention to the downregulated genes and found that two types of genes were obviously downregulated under the action of SADS-CoV nsp1 and FIPV nsp1. The first group contains IFN-related genes, including ISG15, SOCS1, IFI6, OAS1, GBP1, EIF2AK2, IFIT2, STAT1, IRF8, CIITA, CXCL10, and IRF9 (Figure 5B), and the second group contains cell cycle-related genes, including CDKN1A, E2F2, ORC1, SFN, MDM2, and TGFB1 (Figure 5C). To verify the RNA sequencing results, we detected these genes by real-time quantitative PCR and found that ISG15, IFR9, STAT1, CDKN1A, and E2F2 were clearly downregulated by SADS-CoV nsp1 and FIPV nsp1 in various cells (Figure 5D–F). Combined with previous research [28], we constructed the successfully mutant critical region of nsp1 inhibiting host gene expression in FIPV, called by FIPV (91–95 sg) (Figure 6A,B). Compared with the effect by FIPV, we found that STAT1, ISG15, and IRF9 genes were significantly upregulated by FIPV (91–95 sg) (Figure 6C). According to the RNA sequencing results, although nsp1 inhibits the host gene synthesis in a broad-spectrum manner, it is specific for IFN-related genes and cell cycle-related genes.

### 3.5. Alphacoronavirus nsp1 Can Regulate IFN-I and the Cell Cycle

**Figure 7:**
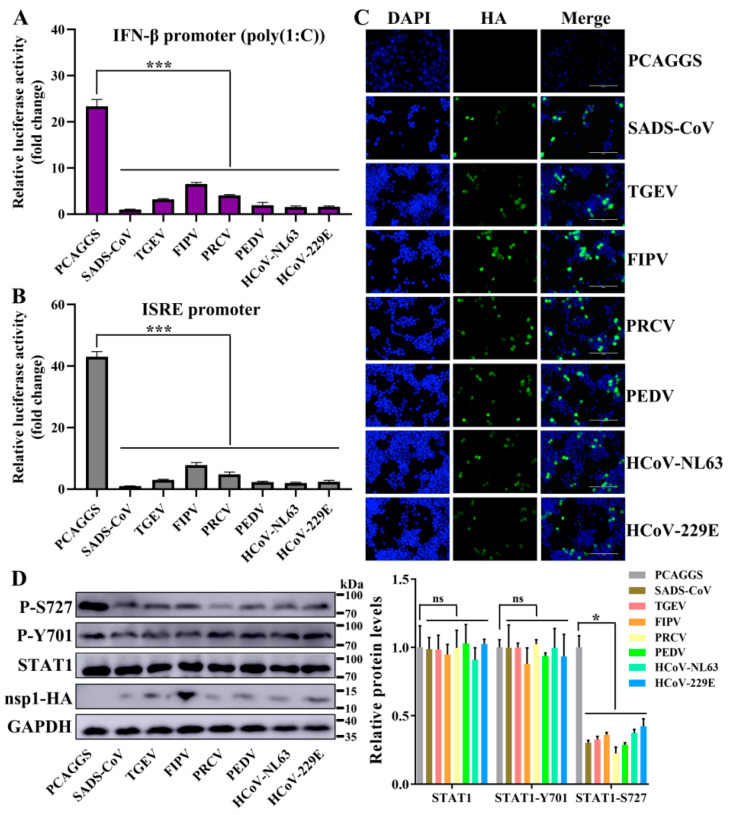
α-CoV nsp1 reduces interferon (IFN)-related gene expression. (**A**,**B**) HEK-293T cells were transfected with a Luc reporter plasmid ([A]; IFN-β promoter, [B]; ISRE promoter) and an expression plasmid encoding a full-length α-CoV nsp1 protein of SADS-CoV, TGEV, FIPV, porcine respiratory coronavirus (PRCV), PEDV, HCoV-NL63 or HcoV-229E. At 8 h post-transfection, the cells were treated with a viral poly(I:C) (A) or IFN-α (B), and the luciferase activity was measured 16 h later. The data are presented as the means ± SD (*n* = 3). Asterisks indicate statistical significance as determined by Student’s *t*-test. ***, *p* < 0.001. (**C**) Transfected HEK-293T cells were fixed at 24 h post-transfection, and indirect immunofluorescence assays were performed with a monoclonal antibody against the HA protein. Original magnification × 200 (scale bars 100 μm). (**D**) Western blot analysis of extracts from HEK-293T cells that were transfected with the mock control or α-CoV nsp1 for 24 h. The analysis was performed with antibodies against STAT1, STAT1 phosphorylation on S727, STAT1 phosphorylation on Y701, HA, and GAPDH (left). The gray scale values of the protein bands were analyzed with ImageJ (right). The data are presented as the means ± SD (*n* = 3). Asterisks indicate statistical significance as determined by Student’s *t*-test. Ns: Not significant; *, *p* < 0.05.

Based on the above-mentioned RNA sequencing results, we wanted to determine whether these two categories have broad-spectrum implications for α-CoV nsp1 proteins. The above-mentioned nsp1 genes in SADS-CoV, PEDV, HCoV-229E, HCoV-NL63, FIPV, TGEV, and PRCV nsp1 were selected for further analysis. To assess the regulatory role of α-CoVs nsp1 in IFN-I signaling, we detected the luciferase activity of α-CoVs nsp1 for the HEK-293T cells that were transfected with the reporter plasmids pRL-IFN-β or pRL-ISRE by the Renilla luciferase (Rluc) assay. The results showed that SADS-CoV, PEDV, HCoV-229E, HCoV-NL63, FIPV, TGEV, and PRCV nsp1 strongly inhibited IFN-α-induced ISRE promoter activity and poly(I:C)-induced IFN-β promoter activity compared with the transcription in the control group (Figure 7A,B). In addition, we confirmed these findings by detecting the expression of α-CoV nsp1 proteins in HEK-293T using the IFA method (Figure 7C). Compared with other α-CoV nsp1s, the ability of FIPV nsp1 to inhibit IFN-β and ISRE reporter genes was greatly decreased. However, there was a higher expression of FIPV nsp1 in HEK-293T cells according to the Western blot analysis (Figure 7D). These results confirm the antagonistic property of α-CoVs nsp1 in IFN-I signaling. Generally, IFN-I signaling induces a potent antiviral state by enhancing the expression of STAT1 phosphorylation [37], which is critical for controlling viral replication. To determine the effectiveness of α-CoV nsp1 on STAT1, we measured the p-STAT1 and total-STAT1 levels in HEK-293T cells. We found that α-CoV nsp1-treated cells markedly downregulated the STAT1 phosphorylation at S727 while having no effect on the total levels of STAT1 and STAT1 phosphorylation on Y701 expression (Figure 7D). This finding validated α-CoV nsp1 as an effective inhibitor of STAT1 phosphorylation at S727.

**Figure 8:**
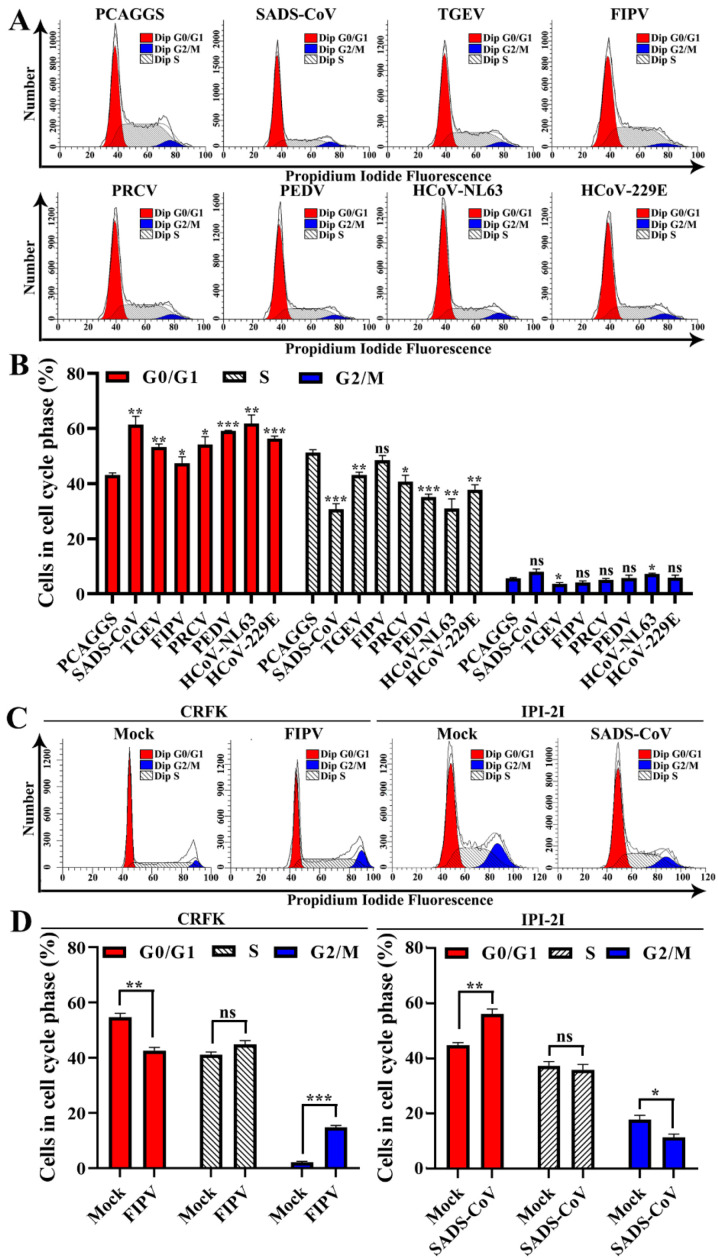
Effects of α-CoV nsp1 on the HEK-293T cell cycle. (**A**) 293T cells transfected with an empty vector or α-CoVs nsp1 vector for 24 h were fixed, stained with PI in the presence of RNase A, and analyzed by flow cytometry to determine the DNA content and the cell population distributions in the various cell cycle phases. (**B**) The statistical result is relative to figure A. (**C**) CRFK cells infected 0.1 MOI FIPV for 12 h or IPI-2I cells infected 0.1 MOI SADS-CoV for 24 h were fixed, stained with PI in the presence of RNase A, and analyzed by flow cytometry to determine the DNA content and the cell population distributions in the various cell cycle phases. (**D**) The statistical result is relative to figure C. Data are represented as means ± SEM (*n* = 3). The values were calculated and differences were considered for *p* < 0.05 (Kruskal Wallis test). Ns: Not significant; *, *p* < 0.05; **, *p* < 0.01; and ***, *p* < 0.001.

To confirm the host cell proliferation inhibition induced by α-CoV nsp1, we measured the cell cycle phase distribution of α-CoV nsp1-treated cells by flow cytometry with propidium iodide (PI) staining. We found that the α-CoV nsp1 treatment induced G0/G1 phase accumulation in HEK-293T cells compared with the control group (Figure 8A,B). Furthermore, the effect of α-CoV 1B nsp1s (SADS-CoV, HCoV-229E, HCoV-NL63, and PEDV nsp1) on cell proliferation inhibition was clearly stronger than that of the α-CoV 1A nsp1s (FIPV, TGEV, and PRCV nsp1). In addition, we had detected the cell cycle of infected cells by SADS-CoV and FIPV. The results showed that SADS-CoV arrest IPI-2I cells in the G0/G1 phase cell cycle, while FIPV induces CRFK cells arrest at the G2/M phase cell cycle (Figure 8C,D), which was consistent with previous reports on PEDV and TGEV [38,39]. Taken together, unlike the complex mechanism by which viruses regulate the cell cycle, α-CoV nsp1 specifically inhibits cell proliferation by arresting in the G0/G1 phase cell cycle.

## 4. Discussion

After the outbreak of SARS-CoV in 2002, MERS-CoV in 2012, and SARS-CoV-2 in 2019 [40,41,42], the continuous emergence of these highly pathogenic viruses has revealed that CoVs have high mutation rates. Furthermore, these novel CoVs appear increasingly more frequently. Currently, the seven types of CoVs prevalent in humans belong to α-CoV and β-CoV [43]. Unfortunately, there is no suitable way to prevent and control α-CoV and β-CoV. Therefore, it is important to design a new vaccine based on basic research about CoV proteins. The nsp1 proteins are present only in α-CoV and β-CoV, with highly variable characteristics [16]. Nsp1 is a very important virulence factor of CoVs. In SARS-CoV, MHV, and TGEV, the virulence of the virus was studied by intercepting the functional region of nsp1 [21,25,26]. It was found that the loss of the ability of nsp1 to inhibit the host protein synthesis would greatly weaken the virulence of the virus in susceptible animals, indicating that nsp1 is a good attenuated vaccine target. In MHV, nsp1 was found to interfere with IFN-I and then affected the virulence of CoV by inhibiting the host protein synthesis. Overall, the virulence of CoV nsp1 is closely related to its function of inhibiting the host protein synthesis. Currently, there are more in-depth studies on the inhibition of host protein synthesis by β-CoV nsp1. SARS-CoV nsp1 blocks the translation of host mRNA by binding to the 40S ribosomal subunit [17]. However, MERS-CoV nsp1 does not bind stably to the 40S ribosomal subunit, but it selectively targets mRNA synthesized in the host nucleus and inhibits host protein translation [20]. These differences also indicate the diversity of functions of β-CoV nsp1 in inhibiting host protein synthesis. In our previous study [21], α-CoV nsp1 was found to regulate protein synthesis through the motif (91–95 aa), but the specific host target of its effect is not very clear. In view of the fact that α-CoV nsp1 has a conserved functional region, we want to explore the general mechanism underlying its inhibition of host protein synthesis.

Here, we are the first to analyze full-length and high-resolution crystal structures from the re-emergent virus SADS-CoV and the highly lethal virus FIPV. Although they belong to different subtypes of α-CoV, their nsp1s both have a conserved fold composed of six β-sheets, which is also present in the PEDV and TGEV nsp1 we previously analyzed [21,22]. These results suggest that the conserved biological function of α-CoV nsp1 depends on this common fold. Although SARS-CoV nsp1 also has this fold, its functional region is not included in the fold but rather is located at the C-terminus, which reflects that there are significant differences in the mechanism of host protein synthesis regulated by nsp1 between α-CoV and β-CoV. We compared the amino acid sequence and tertiary structure of nsp1s from different subtypes and the same subtype in α-CoV, and we found that the nsp1 of the same α-CoV subtype not only had high homology in terms of its amino acid sequence, but it also had a more conserved tertiary structure. Although the secondary structures of nsp1 from different subtypes of α-CoV are similar, there are still significant differences in the loop regions. Among the four α-CoV nsp1s, PEDV nsp1 has a unique feature in that the C-terminus can be observed clearly, while the electron cloud at the C-terminal end of the other α-CoV nsp1 is not clear. By analyzing the structure of PEDV nsp1, we found that its C-terminus reaches a stable state through the action of β4. These differences explain why the key region through which PEDV nsp1 regulates host protein synthesis is different from other α-CoV nsp1s [21,22]. Briefly, we determined that α-CoV nsp1 has a conserved functional fold, and the same subtype of α-CoV nsp1 has a more conserved relationship. We then selected FIPV and SADS-CoV nsp1 as models to explore the general mechanism through which α-CoV nsp1 regulates host protein synthesis. Through the RNA sequencing of mammalian cells treated with FIPV and SADS-CoV nsp1, we found that the two nsp1s could regulate many host genes. A cluster analysis of these affected genes showed that there were 480 common downregulated genes and 493 common upregulated genes. Among the upregulated genes, many metabolism-related genes, such as ND4L, ND4, ND5, ND2, and ND3, may be due to the stress effects produced by the host under the regulation of nsp1 [44,45]. Through bioinformatics analysis, the downregulated genes of SADS-CoV nsp1 and the upregulated genes of FIPV nsp1 were cross-analyzed, and only the AKR1C1 gene was found. Similarly, the upregulated genes of SADS-CoV nsp1 and the downregulated genes of FIPV nsp1 were cross-analyzed, and ARG1, Interleukin 4 induced 1, PLET1, POU2F2, PTGS2, and S100A9 genes were found. It was considered that the function of these genes, Interleukin 4 induced 1 and S100A9 played a prominent role in the regulation of inflammatory processes and immune response, which might have a relevant role in specific pathogenesis of SADS-CoV and FIPV. Since the biological function of nsp1 is primarily regulated by inhibiting the host protein synthesis, we focused on the downregulated genes and found that IFN genes and cell cycle genes are clearly clustered, which reflects that the regulatory functions of the two α-CoV nsp1s may be focused on these two regions. We then verified that seven representative α-CoV nsp1s could significantly inhibit the expression of STAT1-S727 and the activity of IFN-I based on the Rluc and Western blot assay. Interestingly, α-CoV nsp1 only selectively inhibits the activity of STAT1-S727, but it does not affect STAT1 and STAT1-Y701. According to previous studies [46,47], STAT1-S727 is related to the synthesis of CREB-binding protein (CBP), and PEDV nsp1 can inhibit the IFN-I effect by inhibiting the synthesis of CBP. Briefly, α-CoV nsp1 may promote CBP degradation and inhibit IFN-I reaction by selectively inhibiting the phosphorylation of STAT1-S727. A flow analysis was then used to detect the effect of α-CoV nsp1 on the cell cycle of the host cells, and we found that although the effect of α-CoV nsp1 was different, it would cause more host cells to stay in the G0/G1 phase. According to the studies of other viruses [48,49,50], the viral protein will be released to regulate the host cell cycle and allow the host cell to stay in the G0/G1 phase, thus forming a favorable environment for virus production. Taken together, α-CoV nsp1 has a broad-spectrum regulation not only in inhibiting IFN-I but also the cell cycle, which may affect the virulence of the virus.

In summary, we analyzed the tertiary structure of two high-resolution α-CoV nsp1s, which greatly improved the crystal structure data and helped us acknowledge the presence of the common fold in α-CoV nsp1. Through the study of different subtypes of α-CoV nsp1, it was finally confirmed that α-CoV nsp1 can inhibit the effect of IFN-I and the cell cycle. Therefore, we can conclude that α-CoV nsp1 may enhance the virulence of the virus by regulating these two functions of the host.

## Figures and Tables

**Table 1:** Data collection and refinement statistics.

	SADS-CoV nsp1	FIPV nsp1
Data collection		
Space group	P2_1_2_1_2_1_	C121
Cell axial lengths (Å)	54.83, 60.37, 84.00	124.42, 86.30, 87.79
Cell angles (°)	90.00, 90.00, 90.00	90.00, 130.75, 90.00
Wavelength	0.97918	0.97918
Resolution range (Å)	29.19–2.10	29.52–1.80
Completeness (%)	100.0 (100.0)	98.2 (97.1)
R_merge_ (last shell)	0.095 (0.985)	0.126 (0.435)
I/σ (last shell)	21.20 (2.0)	15.27 (3.0)
Redundancy (last shell)	12.9 (13.2)	6.9 (6.7)
Refinement		
Resolution (Å)	29.19–2.10	29.52–1.80
R_work_/R_free_	0.235/0.253	0.187/0.207
No. reflections	16,677	63,361
No. of protein atoms	1560	3360
No. of solvent atoms	69	510
No. of ions/ligand	0	0
RMSD		
Bond length (Å)	0.010	0.007
Bond angle (°)	0.865	0.868
B factor (Å^2^)	56.48	25.76
Ramachandran plot core, allowed, disallowed	98.45%, 1.55%, 0.00%	98.77%, 1.23%, 0.00%

The highest-resolution values are indicated in parentheses. R_merge_ = ∑∑|Ii − <I>|/∑∑Ii, where Ii is the intensity measurement of reflection h, and <I> is the average intensity from multiple observations. R_work_ = ∑||Fo| − |Fc||/∑|Fo|, where Fo and Fc are the observed and calculated structure factors, respectively. Rfree is equivalent to Rwork, but 5% of the measured reflections have been excluded from the refinement and set aside for cross-validation.

**Table 2:** The sequences of primers used for real-time PCR.

Gene	F Primer Sequence (5′ to 3′)	R Primer Sequence (5′ to 3′)	Concentration (μM)	Amplicon Sizes (bp)
H-STAT1	CAGACCACAGACAACCTGCT	ACAGAGCCCACTATCCGAGA	10	112
P-STAT1	GGGCTCTGCTAAAGGACCAG	AGTAAGGTTCGCCTCCGTTC	10	120
F-STAT1	GGGAACCTTACTTCCACGCA	TCAGCCGCCATGACTTTGTA	10	100
H-ISG15	GCGCAGATCACCCAGAAGAT	GTTCGTCGCATTTGTCCACC	10	167
P-ISG15	CTATGAGGTCTGGCTGACGC	GGCTTGAGGTCATACTCCCC	10	147
F-ISG15	GCAGATCGCCCAGAAAACTG	GACCCTTGTGGTTCCTCACC	10	185
H-E2F2	TGCCCAGCTACTGCTACCTA	ATCCCCTCCAGATCCAGCTT	10	154
P-E2F2	GTACCCGCAGACTATGCCTC	AATGCACTTCCCCTTGGGAG	10	187
F-E2F2	GATCAGTTTCTCCCCGCCTT	AGTTAATCAGCAGGTCCCCG	10	113
H-CDKN1A	ATGTGGACCTGTCACTGTCT	CGTTTGGAGTGGTAGAAATCTGTC	10	189
P-CDKN1A	ATGACCTGGGAGGGGGC	GCACAAGGGTACAAGACAGC	10	105
F-CDKN1A	GGAGCGATGGAACTTCGACT	GGGAGTGAGGCATGAGAGTG	10	232
H-IRF9	ACCAGGATGCTGCCTTCTTC	CCTGGTGGCAGCAACTGATA	10	268
P-IRF9	CCCTGCCATCTGGAAGACTC	CCCATTGGTCTCTGCCAACT	10	289
F-IRF9	TTCTTCAAGGCATGGGCGAT	TTTGTGGTACCCGCATCCTC	10	278
H-GAPDH	CAAATTCCATGGCACCGTCA	GACTCCACGACGTACTCAGC	10	211
P-GAPDH	TCGGAGTGAACGGATTTGGC	TCTCGCTCCTGGAAGATGGT	10	226
F-GAPDH	AAGGTCGGTGTGAACGGATT	TTTGCCGTGGGTGGAATCAT	10	152

## References

[1] Jiang S., Du L., Shi Z. (2020). An emerging coronavirus causing pneumonia outbreak in wuhan, china: Calling for developing therapeutic and prophylactic strategies. Emerg. Microbes Infect..

[2] Weiss S.R., Leibowitz J.L. (2011). Coronavirus pathogenesis. Adv. Virus Res..

[3] Fehr A.R., Perlman S. (2015). Coronaviruses: An overview of their replication and pathogenesis. Methods Mol. Biol..

[4] Belouzard S., Millet J.K., Licitra B.N., Whittaker G.R. (2012). Mechanisms of coronavirus cell entry mediated by the viral spike protein. Viruses.

[5] Perlman S., Netland J. (2009). Coronaviruses post-sars: Update on replication and pathogenesis. Nat. Rev. Microbiol..

[6] Yin Y.D., Wunderink R.G. (2018). Mers, sars and other coronaviruses as causes of pneumonia. Respirology.

[7] Whittaker G.R., Andre N.M., Millet J.K. (2018). Improving virus taxonomy by recontextualizing sequence-based classification with biologically relevant data: The case of the alphacoronavirus 1 species. mSphere.

[8] Zhou P., Fan H., Lan T., Yang X.L., Shi W.F., Zhang W., Zhu Y., Zhang Y.W., Xie Q.M., Mani S. (2018). Fatal swine acute diarrhoea syndrome caused by an hku2-related coronavirus of bat origin. Nature.

[9] Li K., Li H., Bi Z., Song D., Zhang F., Lei D., Luo S., Li Z., Gong W., Huang D. (2019). Significant inhibition of re-emerged and emerging swine enteric coronavirus in vitro using the multiple shrna expression vector. Antivir. Res..

[10] Zhou L., Li Q.N., Su J.N., Chen G.H., Wu Z.X., Luo Y., Wu R.T., Sun Y., Lan T., Ma J.Y. (2019). The re-emerging of sads-cov infection in pig herds in southern china. Transbound. Emerg. Dis..

[11] Yang Y.L., Qin P., Wang B., Liu Y., Xu G.H., Peng L., Zhou J., Zhu S.J., Huang Y.W. (2019). Broad cross-species infection of cultured cells by bat hku2-related swine acute diarrhea syndrome coronavirus and identification of its replication in murine dendritic cells in vivo highlight its potential for diverse interspecies transmission. J. Virol..

[12] Pedersen N.C. (2014). An update on feline infectious peritonitis: Virology and immunopathogenesis. Vet. J..

[13] Kipar A., Meli M.L. (2014). Feline infectious peritonitis: Still an enigma?. Vet. Pathol..

[14] Yang Y.L., Liang Q.Z., Xu S.Y., Mazing E., Xu G.H., Peng L., Qin P., Wang B., Huang Y.W. (2019). Characterization of a novel bat-hku2-like swine enteric alphacoronavirus (seacov) infection in cultured cells and development of a seacov infectious clone. Virology.

[15] Woo P.C., Huang Y., Lau S.K., Yuen K.Y. (2010). Coronavirus genomics and bioinformatics analysis. Viruses.

[16] Snijder E.J., Bredenbeek P.J., Dobbe J.C., Thiel V., Ziebuhr J., Poon L.L., Guan Y., Rozanov M., Spaan W.J., Gorbalenya A.E. (2003). Unique and conserved features of genome and proteome of sars-coronavirus, an early split-off from the coronavirus group 2 lineage. J. Mol. Biol..

[17] Kamitani W., Huang C., Narayanan K., Lokugamage K.G., Makino S. (2009). A two-pronged strategy to suppress host protein synthesis by sars coronavirus nsp1 protein. Nat. Struct. Mol. Biol..

[18] Tanaka T., Kamitani W., DeDiego M.L., Enjuanes L., Matsuura Y. (2012). Severe acute respiratory syndrome coronavirus nsp1 facilitates efficient propagation in cells through a specific translational shutoff of host mrna. J. Virol..

[19] Nakagawa K., Narayanan K., Wada M., Popov V.L., Cajimat M., Baric R.S., Makino S. (2018). The endonucleolytic rna cleavage function of nsp1 of middle east respiratory syndrome coronavirus promotes the production of infectious virus particles in specific human cell lines. J. Virol..

[20] Lokugamage K.G., Narayanan K., Nakagawa K., Terasaki K., Ramirez S.I., Tseng C.T., Makino S. (2015). Middle east respiratory syndrome coronavirus nsp1 inhibits host gene expression by selectively targeting mrnas transcribed in the nucleus while sparing mrnas of cytoplasmic origin. J. Virol..

[21] Shen Z., Wang G., Yang Y., Shi J., Fang L., Li F., Xiao S., Fu Z.F., Peng G. (2019). A conserved region of nonstructural protein 1 from alphacoronaviruses inhibits host gene expression and is critical for viral virulence. J. Biol. Chem..

[22] Shen Z., Ye G., Deng F., Wang G., Cui M., Fang L., Xiao S., Fu Z.F., Peng G. (2018). Structural basis for the inhibition of host gene expression by porcine epidemic diarrhea virus nsp1. J. Virol..

[23] Almeida M.S., Johnson M.A., Wuthrich K. (2006). NMR assignment of the sars-cov protein nsp1. J. Biomol. NMR.

[24] Huang C., Lokugamage K.G., Rozovics J.M., Narayanan K., Semler B.L., Makino S. (2011). Alphacoronavirus transmissible gastroenteritis virus nsp1 protein suppresses protein translation in mammalian cells and in cell-free hela cell extracts but not in rabbit reticulocyte lysate. J. Virol..

[25] Zust R., Cervantes-Barragan L., Kuri T., Blakqori G., Weber F., Ludewig B., Thiel V. (2007). Coronavirus non-structural protein 1 is a major pathogenicity factor: Implications for the rational design of coronavirus vaccines. PLoS Pathog.

[26] Wathelet M.G., Orr M., Frieman M.B., Baric R.S. (2007). Severe acute respiratory syndrome coronavirus evades antiviral signaling: Role of nsp1 and rational design of an attenuated strain. J. Virol..

[27] Narayanan K., Huang C., Lokugamage K., Kamitani W., Ikegami T., Tseng C.T., Makino S. (2008). Severe acute respiratory syndrome coronavirus nsp1 suppresses host gene expression, including that of type i interferon, in infected cells. J. Virol..

[28] Wang G., Liang R., Liu Z., Shen Z., Shi J., Shi Y., Deng F., Xiao S., Fu Z.F., Peng G. (2019). The n-terminal domain of spike protein is not the enteric tropism determinant for transmissible gastroenteritis virus in piglets. Viruses.

[29] Dong N., Fang L., Yang H., Liu H., Du T., Fang P., Wang D., Chen H., Xiao S. (2016). Isolation, genomic characterization, and pathogenicity of a chinese porcine deltacoronavirus strain chn-hn-2014. Vet. Microbiol..

[30] Ye G., Deng F., Shen Z., Luo R., Zhao L., Xiao S.B., Fu Z.F., Peng G.Q. (2016). Structural basis for the dimerization and substrate recognition specificity of porcine epidemic diarrhea virus 3c-like protease. Virology.

[31] Emsley P., Cowtan K. (2004). Coot: Model-building tools for molecular graphics. Acta Crystallogr. D Biol. Crystallogr..

[32] Adams P.D., Grosse-Kunstleve R.W., Hung L.W., Ioerger T.R., McCoy A.J., Moriarty N.W., Read R.J., Sacchettini J.C., Sauter N.K., Terwilliger T.C. (2002). Phenix: Building new software for automated crystallographic structure determination. Acta Crystallogr. D Biol. Crystallogr..

[33] Grell L., Parkin C., Slatest L., Craig P.A. (2006). Ez-viz, a tool for simplifying molecular viewing in pymol. Biochem. Mol. Biol. Educ..

[34] Tamura K., Peterson D., Peterson N., Stecher G., Nei M., Kumar S. (2011). Mega5: Molecular evolutionary genetics analysis using maximum likelihood, evolutionary distance, and maximum parsimony methods. Mol. Biol. Evol..

[35] Lv G.Y., Sun D.J., Zhang J.W., Xie X.X., Wu X.Q., Fang W.S., Tian J.W., Yan C.H., Wang H.B., Fu F.H. (2017). Lx2-32c, a novel semi-synthetic taxane, exerts antitumor activity against prostate cancer cells in vitro and in vivo. Acta Pharm. Sin. B.

[36] Cheng V.C., Lau S.K., Woo P.C., Yuen K.Y. (2007). Severe acute respiratory syndrome coronavirus as an agent of emerging and reemerging infection. Clin. Microbiol. Rev..

[37] Zhang S.P., Huo C.Y., Xiao J., Fan T., Zou S.M., Qi P., Sun L.Q., Wang M., Hu Y.X. (2019). P-stat1 regulates the influenza a virus replication and inflammatory response in vitro and vivo. Virology.

[38] Ding L., Huang Y., Dai M.L., Zhao X.M., Du Q., Dong F., Wang L.L., Huo R.C., Zhang W.L., Xu X.G. (2013). Transmissible gastroenteritis virus infection induces cell cycle arrest at s and g2/m phases via p53-dependent pathway. Virus Res..

[39] Luo Y.R., Zhou S.T., Yang L., Liu Y.P., Jiang S.Y., Dawuli Y., Hou Y.X., Zhou T.X., Yang Z.B. (2020). Porcine epidemic diarrhoea virus induces cell-cycle arrest through the DNA damage-signalling pathway. J. Vet. Res..

[40] Guan Y., Zheng B.J., He Y.Q., Liu X.L., Zhuang Z.X., Cheung C.L., Luo S.W., Li P.H., Zhang L.J., Guan Y.J. (2003). Isolation and characterization of viruses related to the sars coronavirus from animals in southern china. Science.

[41] Zaki A.M., van Boheemen S., Bestebroer T.M., Osterhaus A.D., Fouchier R.A. (2012). Isolation of a novel coronavirus from a man with pneumonia in saudi arabia. N. Engl. J. Med..

[42] Wang C., Horby P.W., Hayden F.G., Gao G.F. (2020). A novel coronavirus outbreak of global health concern. Lancet.

[43] Rabaan A.A., Al-Ahmed S.H., Haque S., Sah R., Tiwari R., Malik Y.S., Dhama K., Yatoo M.I., Bonilla-Aldana D.K., Rodriguez-Morales A.J. (2020). Sars-cov-2, sars-cov, and mers-cov: A comparative overview. Infez. Med..

[44] Nelson M.A., Macino G. (1987). Structure and expression of the overlapping nd4l and nd5 genes of neurospora crassa mitochondria. Mol. Gen. Genet..

[45] Cardol P., Lapaille M., Minet P., Franck F., Matagne R.F., Remacle C. (2006). Nd3 and nd4l subunits of mitochondrial complex i, both nucleus encoded in chlamydomonas reinhardtii, are required for activity and assembly of the enzyme. Eukaryot. Cell.

[46] Pilz A., Ramsauer K., Heidari H., Leitges M., Kovarik P., Decker T. (2003). Phosphorylation of the stat1 transactivating domain is required for the response to type i interferons. EMBO Rep..

[47] Zhang Q., Shi K., Yoo D. (2016). Suppression of type i interferon production by porcine epidemic diarrhea virus and degradation of creb-binding protein by nsp1. Virology.

[48] Davies C., Brown C.M., Westphal D., Ward J.M., Ward V.K. (2015). Murine norovirus replication induces g0/g1 cell cycle arrest in asynchronously growing cells. J. Virol..

[49] Yuan X., Shan Y., Zhao Z., Chen J., Cong Y. (2005). G0/g1 arrest and apoptosis induced by sars-cov 3b protein in transfected cells. Virol. J..

[50] Yuan X.L., Wu J., Shan Y.J., Yao Z.Y., Dong B., Chen B., Zhao Z.H., Wang S.Q., Chen J.P., Cong Y.W. (2006). Sars coronavirus 7a protein blocks cell cycle progression at g0/g1 phase via the cyclin d3/prb pathway. Virology.

